# Changes in abdominal subcutaneous adipose tissue phenotype following menopause is associated with increased visceral fat mass

**DOI:** 10.1038/s41598-021-94189-2

**Published:** 2021-07-20

**Authors:** Julie Abildgaard, Thorkil Ploug, Elaf Al-Saoudi, Thomas Wagner, Carsten Thomsen, Caroline Ewertsen, Michael Bzorek, Bente Klarlund Pedersen, Anette Tønnes Pedersen, Birgitte Lindegaard

**Affiliations:** 1 The Centre of Inflammation and Metabolism and the Centre for Physical Activity Research, Rigshospitalet, University of Copenhagen, Blegdamsvej 9, 2100 Copenhagen, Denmark; 2 Department of Growth and Reproduction, Copenhagen University Hospital, Rigshospitalet, Copenhagen, Denmark; 3 Department of Biomedical Sciences, Faculty of Health and Medical Sciences, University of Copenhagen, Copenhagen, Denmark; 4 Department of Oncology, Copenhagen University Hospital, Rigshospitalet, Copenhagen, Denmark; 5 Department of Radiology, Zealand University Hospital, Roskilde, Denmark; 6 Department of Radiology, Copenhagen University Hospital, Rigshospitalet, Copenhagen, Denmark; 7 Department of Pathology, Zealand University Hospital, Næstved, Denmark; 8 Department of Gynecology, Copenhagen University Hospital, Rigshospitalet, Copenhagen, Denmark; 9 Department of Pulmonary and Infectious Diseases, Nordsjællands University Hospital, Hillerød, Denmark

**Keywords:** Metabolic syndrome, Pre-diabetes, Hypogonadism, Risk factors, Molecular medicine

## Abstract

Menopause is associated with a redistribution of adipose tissue towards central adiposity, known to cause insulin resistance. In this cross-sectional study of 33 women between 45 and 60 years, we assessed adipose tissue inflammation and morphology in subcutaneous adipose tissue (SAT) and visceral adipose tissue (VAT) across menopause and related this to menopausal differences in adipose tissue distribution and insulin resistance. We collected paired SAT and VAT biopsies from all women and combined this with anthropometric measurements and estimated whole-body insulin sensitivity. We found that menopause was associated with changes in adipose tissue phenotype related to metabolic dysfunction. In SAT, postmenopausal women showed adipocyte hypertrophy, increased inflammation, hypoxia and fibrosis. The postmenopausal changes in SAT was associated with increased visceral fat accumulation. In VAT, menopause was associated with adipocyte hypertrophy, immune cell infiltration and fibrosis. The postmenopausal changes in VAT phenotype was associated with decreased insulin sensitivity. Based on these findings we suggest, that menopause is associated with changes in adipose tissue phenotype related to metabolic dysfunction in both SAT and VAT. Whereas increased SAT inflammation in the context of menopause is associated with VAT accumulation, VAT morphology is related to insulin resistance.

## Introduction

Loss of ovarian function leads to an increased risk of metabolic disease including metabolic syndrome, type 2 diabetes, and cardiovascular disease^1–3^. The mechanisms are poorly understood but is believed to be related to the increased visceral adipose tissue (VAT) deposition seen in relation to menopause^4–6^. For a given total fat mass, postmenopausal women store significantly more VAT than premenopausal women^4^. The mechanisms behind the redistribution of fat with menopause are largely unknown. However, it is generally believed that subcutaneous adipose tissue (SAT) dysfunction, e.g. as seen with lipodystrophy^7^, cause lipid spill over from SAT, whereby lipids will be redirected and stored in less favorable anatomic locations such as the VAT (and other ectopic sites)^8,9^.

We previously showed, that an increase in both total fat mass and VAT mass were associated with a more pronounced increase in ectopic lipid deposition and insulin resistance (IR) in postmenopausal women compared with premenopausal women^4^. These findings indicate that the health-related consequences linked to expanding the fat mass might increase after menopause and suggest that postmenopause could be related to AT dysfunction, both in SAT and VAT, with a lower threshold for lipid spillover to peripheral organs, in postmenopausal women.

The manner in which the adipose tissue (AT) expands and remodels, directly impacts the risk of metabolic disease^10,11^. Pathologic AT remodeling is characterized by adipocyte hypertrophy, pericellular fibrosis (PcF), and chronic inflammation all contributing to IR^12–15^. Studies in rodents show that oophorectomy leads to an increased adipocyte size, infiltration of immune cells, and a higher expression of inflammatory cytokines in both inguinal- and gonadal adipose depots (equivalent to SAT and VAT in humans)^16–20^. In humans, postmenopause is associated with systemic low-grade inflammation^21^. However, little is known about the relationship between menopause, changes in AT phenotype and metabolic disease.

We collected paired SAT and VAT biopsies from pre-, peri-, and postmenopausal women. We hypothesized, that menopause was associated with changes in AT phenotype, and that this could be related to postmenopausal AT redestribution and IR.

## Results

### Subject characteristics

Subject characteristics are shown in Table 1.

**Table 1:** Subject characteristics.

	Premenopausal	Perimenopausal	Postmenopausal	p-value
N	13	5	15	
Age, years	48.7 (45.5–50.0)	53.4 (53.0–53.7)	54.3 (51.7–56.9)	< 0.001
**Gynecological surgery**				
Hysterectomy	7	5	9	
Unilateral salpingo-oophorectomy	4	0	4	
Bilateral sapingo-oophorectomy	2	0	2	
**Sex hormones**				
E_2_, nmol/L	0.31 (0.17–0.53)	0.09^a^ (0.09–0.09)	0.09^a^ (0.09–0.18)	0.005
FSH, IU/L	6.7 (4.3–8.5)	61.3^a^ (53.3–71.8)	62.6^a^ (42.1–76.0)	< 0.001
**Body composition**				
Body weight, kg	68.6 (59.1–72.6)	65.1 (60.2–70.0)	70.1 (63.1–81.9)	0.55
Height, m	1.71 (1.68–1.78)	1.65 (1.62–1.71)	1.68 (1.64–1.74)	0.10
BMI, kg/m^2^	22.5 (20.1–24.6)	23.9 (23.9–24.4)	25.5 (22.1–27.6)	0.13
Lean body mass, kg	42.1 (40.2–45.6)	40.6 (37.4–41.7)	39.5 (39.1–42.8)	0.38
Total fat mass, kg	22.9 (15.7–23.3)	24.4 (20.1–24.6)	25.1 (22.5–38.6)	0.12
Body fat, %	32 (27–33)	35 (33–37)	38^a^ (35–47)	0.02
Trunk fat, kg	9.5 (7.3–11.1)	12.0 (8.9–21.9)	12.9 (9.5–22.5)	0.10
Limb fat, kg	10.8 (8.9–12.1)	9.5 (7.1–14.1)	12.3 (10.0–15.0)	0.23
Trunk:limb fat ratio, AU	0.93 (0.76–1.12)	1.34 (0.93–1.80)	1.12 (1.01–1.25)	0.07
Abdominal SAT, cm^2^	160.4 (134.3–228.6)	214.7 (163.9–227.5)	248.2^a^ (196.4–299.1)	< 0.05
VAT mass, L	0.35 (0.28–0.62)	0.69 (0.40–1.91)	1.21^a^ (0.70–1.98)	0.01
**Exercise capacity**				
VO_2_ max, mL/min	2160 (2026–2508)	1862 (1700–2100)	1615^a^ (1434–2046)	0.004
VO_2_ max/lean body mass, mL/kg lean mass/min	53.3 (51.2–58.0)	45.9 (39.0–57.2)	43.6^a^ (36.7–49.1)	0.02
**Physical activity**^b^				
Total METs	413 (342–516)	351 (198–768)	272 (192–335)	0.29
**Energy intake**^b^				
Total intake, kJ	1701 (1664–2003)	1763 (1699–2066)	1859 (1640–2261)	0.95
Carbohydrate, g	178 (149–183)	209 (153–247)	223 (163–255)	0.16
Protein, g	64 (59–84)	69 (67–91)	72 (66–77)	0.67
Fat, g	76 (63–92)	67 (54–104)	75 (59–97)	0.91
**Plasma lipids**				
Total cholesterol, mmol/L	4.8 (4.3–5.6)	5.9 (5.3–6.0)	5.6 (5.0–5.8)	0.11
HDL cholesterol, mmol/L	1.7 (1.4–2.0)	1.4 (1.3–2.1)	1.5 (1.4–1.9)	0.78
LDL cholesterol, mmol/L	2.6 (2.3–3.4)	4.0 (3.7–4.0)	3.7 (3.4–4.0)	0.13
Triglycerides, mmol/L	0.89 (0.80–0.95)	1.16 (0.86–1.74)	1.05 (0.78–1.36)	0.13
FFA, mmol/L	0.47 (0.34–0.54)	0.46 (0.30–0.63)	0.49 (0.28–0.57)	0.89
Glycerol, mmol/L	0.08 (0.05–0.09)	0.12 (0.06–0.21)	0.08 (0.06–0.10)	0.09
**Glucose metabolism**				
Fasting glucose, mmol/L	4.7 (4.5–5.0)	5.6 (5.3–5.9)	5.0 (4.7–5.2)	0.06
Fasting insulin, pmol/L	43 (26–58)	80 (48–85)	53 (44–68)	0.27
HbA1c, mmol/L	5.7 (5.4–5.8)	6.1^a^ (5.8–6.8)	6.1^a^ (6.0–6.9)	0.02
Glucose AUC, AU	737 (713–875)	1094^a^ (823–1273)	1064^a^ (815–1184)	0.03
Insulin AUC, AU	34,076 (27,761–48,435)	62,970^a^ (47,801–83,752)	53,43^a^ (36,113–81,750)	0.04
Estimated insulin sensitivity^c^	6.7 (4.9–11.3)	2.7^a^ (2.3–4.8)	4.6^a^ (2.7–6.5)	0.02

Data are presented as median (interquartile range).

*E*_*2*_ estradiol, *FSH* follicle stimulating hormone, *IU* international units, *AU* arbitrary units, *BMI* body mass index, *SAT* subcutaneous adipose tissue, *VAT* Visceral adipose tissue, *L* liter, *VO*_*2*_* max* maximum oxygen uptake, *MET* Metabolic equivalents, *LDL* low-density lipoprotein, *HDL* high-density lipoprotein, *FFA* free fatty acids, *AUC* area under the curve.

^a^Significantly different from premenopausal, p < 0.05.

^b^Self-reported.

^c^Estimated insulin sensitivity calculated through Composite Matsuda Index.

Thirteen women were defined as premenopausal, five as perimenopausal, and fifteen as postmenopausal. Postmenopausal women had a median time since menopause of 4 (interquartile range, IQR 3–7) years.

The groups showed no significant differences in body weight (p = 0.55) or total fat mass (p = 0.12). Postmenopausal women had a 123% (95% CI 16–231%) bigger VAT mass compared to premenopausal women and for a given total fat mass, postmenopausal women stored 18% (95% CI 2–33%) more VAT.

Peri- and postmenopausal women had higher plasma HbA1c (peri: 12%, 95% CI 1–23% and post: 11%, 95% CI 3–19%) and a lower estimated insulin sensitivity (IS) (peri: 42%, 95% CI 7–77% and post: 29%, 95% CI 3–55%) compared to premenopausal women.

### Adipocyte size and number

**Figure 1:**
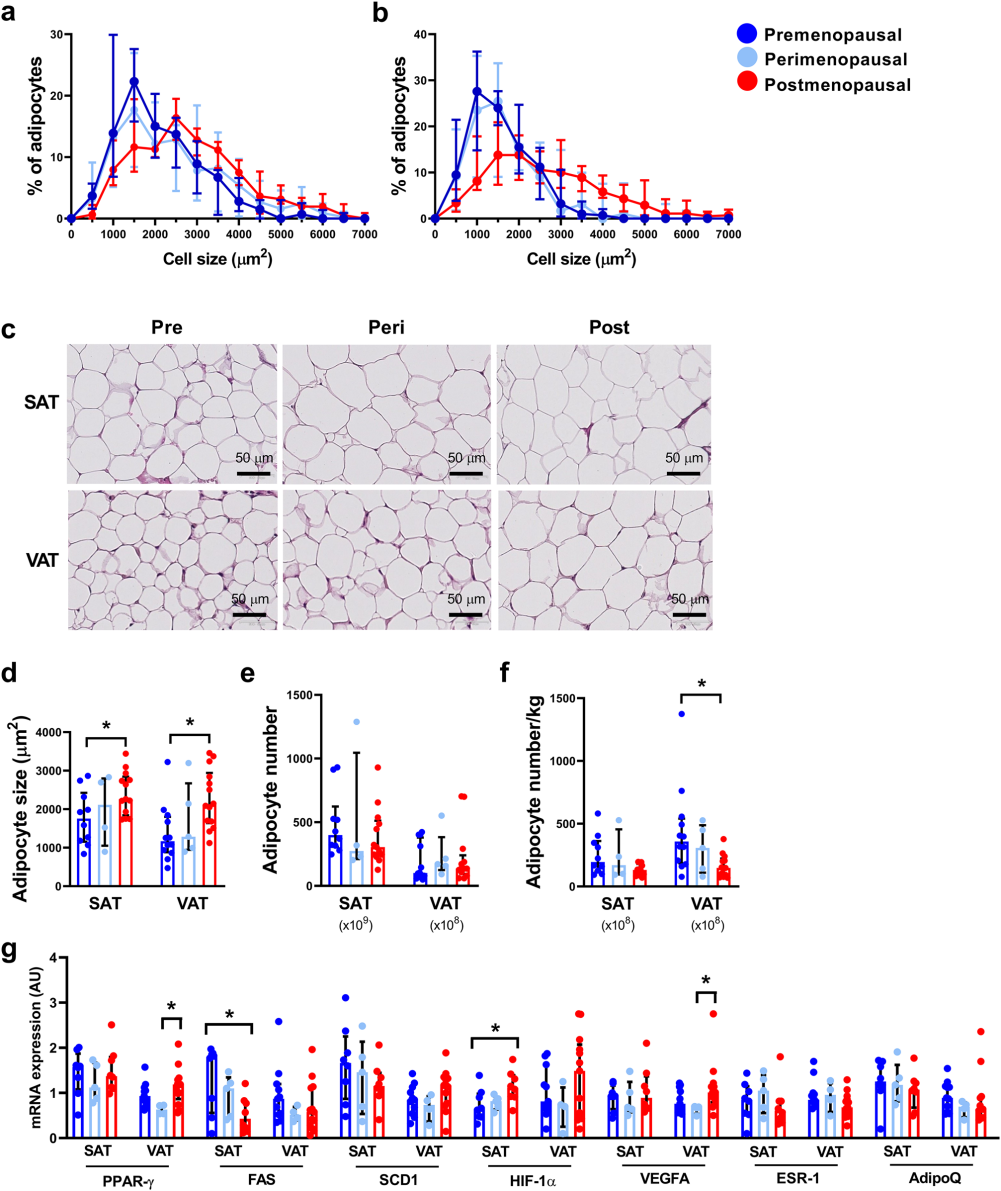
Adipocyte size and mRNA expression in subcutaneous adipose tissue (SAT) and visceral adipose tissue (VAT) in pre- (SAT, n = 10; VAT, n = 12), peri- (SAT and VAT, n = 5), and postmenopausal women (SAT, n = 12; VAT, n = 14). (**A**) Adipocyte size distribution in SAT in pre-, peri-, and postmenopausal women, (**B**) Adipocyte size distribution in VAT, (**C**) Representative HE-section of SAT and VAT, (**D**) Adipocyte size in SAT and VAT, (**E**) Adipocyte number in SAT and VAT, (**F**) Adipocyte number per kg fat in SAT and VAT, (**G**) mRNA expression of Peroxisome Proliferator-Activated Receptor (PPAR)-γ, Fatty Acid Synthase (FAS), Steraoyl-CoA Desaturase (SCD)1, Hypoxia-inducible factor (HIF)-1α, Vascular Endothelial Growth Factor A (VEGFA), Estrogen Receptor 1 (ESR1), and Adiponectin (AdipoQ) in both SAT and VAT. Data are presented as median (interquartile range). *Significant difference, p < 0.05.

Adipocyte size distribution differed significantly between pre- and postmenopausal women in both SAT (p < 0.01) and VAT (p < 0.01) with a higher frequency of smaller adipocytes in premenopausal women and of bigger adipocytes in postmenopausal women. Adipocyte size distribution in perimenopausal women was not significantly different from either pre- or postmenopausal women (Fig. 1A–D). Menopausal status did not affect total number of adipocytes in SAT (p = 0.31) or VAT (p = 0.86) (Fig. 1E). Adipocyte number per kg SAT was only borderline associated with menopausal status with a tendency to fewer adipocytes after menopause (p = 0.07). Postmenopausal women had 15% (95% CI 5–24%) fewer adipocytes per kg VAT compared to premenopausal women (Fig. 1F).

Messenger-RNA (mRNA) expression of the adipogenic marker Peroxisome proliferator activated receptor (PPAR)-γ in SAT was not associated with menopausal status (p = 0.60). PPAR-γ in VAT was increased (83%, 95% CI 21–144%) in postmenopausal women compared to perimenopausal women. Fatty acid synthase (FAS), a marker of lipogenesis, was decreased by 61% (95% CI 16–106%) in SAT in postmenopausal women compared to premenopausal women with no difference in VAT (p = 0.26). Stearoyl-CoA desaturase (SCD)1, reflecting monounsaturated lipogenesis, was not associated with menopausal status in either SAT (p = 0.48) or VAT (p = 0.19). In SAT, hypoxia inducible factor (HIF)-1α expression was significantly increased (255%, 95% CI 87–394%) in postmenopausal women compared to premenopausal women. VAT HIF-1α was not associated with menopausal status (p = 0.27). SAT mRNA expression of Vascular endothelial growth factor A (VEGFA) was not associated with menopausal status (p = 0.69). VAT VEGFA mRNA expression was significantly increased in postmenopausal women compared to perimenopausal women (105%, 95% CI 15–195%). mRNA expression of estrogen receptor 1 (ESR1) was equally distributed across menopausal status and fat depots as was adiponectin (Fig. 1G).

### Adipose tissue immune cell infiltration

**Figure 2:**
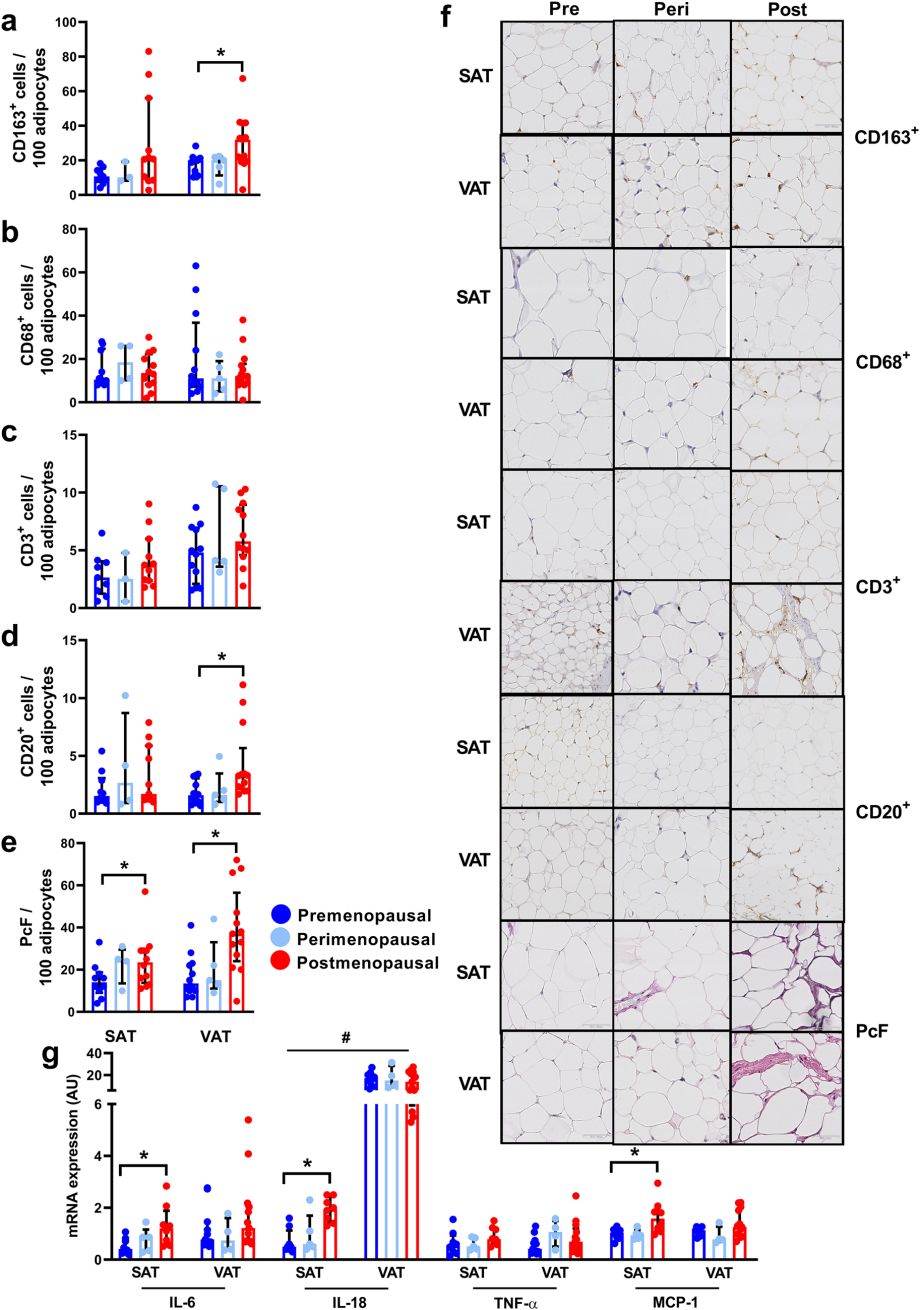
Morphology and inflammation in subcutaneous adipose tissue (SAT) and visceral adipose tissue (VAT) in pre- (SAT, n = 10; VAT, n = 12), peri- (SAT and VAT, n = 5), and postmenopausal women (SAT, n = 12; VAT, n = 14). (**A**) Number of CD163^+^ cells/100 adipocytes in SAT and VAT in pre-, peri-, and postmenopausal women, (**B**) Number of CD68^+^ cells/100 adipocytes in SAT and VAT, (**C**) Number of CD3^+^ cells/100 adipocytes in SAT and VAT, (**D**) Number of CD20^+^ cells/100 adipocytes in SAT and VAT, (**E**) Pericellular fibrosis (PcF)/100 adipocytes in SAT and VAT, (**F**) Representative immunohistochemistry- and counter stainings of SAT and VAT, (**G**) mRNA expression of interleukin (IL)-6, IL-18, Tumor Necrosis Factor (TNF)-α, and Monocyte Chemoattractant Protein (MCP)-1, in SAT and VAT. Data are presented as median (interquartile range). *Significant difference, p < 0.05. ^#^Significant difference between SAT and VAT, p < 0.05.

CD163^+^ cell infiltration in SAT was not significantly associated with menopausal status (p = 0.11). In VAT, number of CD163^+^ cells were increased by 111% (95% CI 26–195%) in postmenopausal women compared to premenopausal women (Fig. 2A). AT CD68^+^ cell infiltration was comparable across menopausal status in both SAT (p = 0.66) and VAT (p = 0.40), as was CD3^+^ cell infiltration (SAT: p = 0.22 and VAT: p = 0.26) (Fig. 2B,C). CD20^+^ cell infiltration in SAT was not significantly associated with menopausal status (p = 0.63). Postmenopausal women had a 169% (95% CI 59–278%) increase in CD20^+^ cells in VAT compared to premenopausal women (Fig. 2D).

### Adipose tissue fibrosis

Postmenopausal women had increased PcF in both SAT (21%, 95% CI 3–40%) and VAT (130%, 95% CI 54–206%) compared to premenopausal women (Fig. 2E,F).

### Adipose tissue inflammatory markers

mRNA expression of interleukin (IL)-6 in SAT was increased by 152% (95% CI 35–267%) in postmenopausal women compared to premenopausal women. VAT IL-6 mRNA expression was not associated with menopausal status (p = 0.34). IL-18 expression in SAT was increased by 41% (95% CI 21–60%) in postmenopausal women compared to premenopausal women. VAT IL-18 mRNA expression was not associated with menopausal status (p = 0.76). IL-18 mRNA expression was 7.6 (95% CI 6.7–8.5) times higher in VAT compared to SAT (p < 0.0001). Tumor necrosis factor (TNF)-α expression in AT was comparable across menopausal status (SAT, p = 0.25 and VAT, p = 0.23). Monocyte Chemoattractant Protein (MCP)-1 expression was increased by 67% (95% CI 20–114%) in SAT in postmenopausal women compared to premenopausal women. VAT MCP-1 mRNA expression was not significantly associated with menopausal status (p = 0.09) (Fig. 2G).

### Subcutaneous adipose tissue phenotype visceral fat accumulation

In this cohort, an increased VAT mass was associated with low SAT FAS (β = − 1.33, p < 0.001), high HIF-1α (β = 0.52, p = 0.01), CD163^+^ (β = 0.09, p = 0.04), CD3^+^ (β = 0.45, p = 0.04), CD20^+^ cell infiltration (β = 0.65, p = 0.02), IL-6 (β = 0.50, p = 0.04), IL-18 (β = 3.28, p = 0.003), TNF-α (β = 0.40, p = 0.03), and MCP-1 mRNA expression (β = 0.93, p = 0.04) after adjusting for total fat mass (Table 2).

**Table 2:** Subcutaneous adipose tissue inflammation and morphology associated with visceral adipose tissue mass.

	VAT mass, L		VAT mass, L^a^	
	β (95% CI)	p-value	β (95% CI)	p-value
SAT Adipocyte size	0.20 (− 0.76 to 1.15)	0.67	− 0.27 (− 1.17 to 0.62)	0.53
SAT PPAR-γ	− 0.29 (− 0.81 to 0.22)	0.24	0.04 (− 0.65 to 0.72)	0.90
SAT FAS	− 1.23 (− 1.67 to − 0.80)	< 0.001	− 1.33 (− 1.93 to − 0.72)	< 0.001
SAT SCD1	− 0.86 (− 1.56 to − 0.17)	0.02	− 0.78 (− 1.75 to 0.20)	0.11
SAT HIF-1α	0.77 (0.34 to 1.21)	0.002	0.52 (0.13 to 0.92)	0.01
SAT VEGFA	− 0.40 (− 0.88 to 0.08)	0.10	− 0.06 (− 0.50 to 0.39)	0.80
SAT ESR1	− 0.37 (− 0.80 to 0.07)	0.09	− 0.03 (− 0.44 to 0.38)	0.88
SAT AdipoQ	− 0.52 (− 0.92 to − 0.12)	0.01	− 0.19 (− 0.61 to 0.24)	0.36
SAT CD163^+^ cells	0.13 (0.07 to 0.20)	< 0.001	0.09 (0.00 to 0.17)	0.04
SAT CD68^+^ cells	0.16 (− 0.04 to 0.35)	0.10	0.14 (− 0.03 to 0.30)	0.10
SAT CD3^+^ cells	0.65 (0.15 to 1.15)	0.01	0.45 (0.03 to 0.86)	0.04
SAT CD20^+^ cells	0.86 (0.28 to 1.44)	0.005	0.65 (0.11 to 1.19)	0.02
SAT PcF	0.17 (0.04 to 0.30)	0.01	0.10 (− 0.04 to 0.24)	0.14
SAT IL-6 mRNA	0.75 (0.19 to 1.30)	0.01	0.50 (0.03 to 0.96)	0.04
SAT IL-18 mRNA	4.34 (2.71 to 5.98)	< 0.001	3.28 (1.23 to 5.33)	0.003
SAT TNF-α mRNA	0.59 (0.15 to 1.04)	0.01	0.40 (0.03 to 0.77)	0.03
SAT MCP-1 mRNA	1.24 (0.09 to 2.39)	0.04	0.93 (0.04 to 1.81)	0.04

*SAT* subcutaneous adipose tissue, *VAT* visceral adipose tissue, *PPAR* Peroxisome proliferator activated receptor, *FAS* fatty-acid synthase, *SCD* Stearoyl-CoA desaturase, *HIF* Hypoxia-inducible factor, *VEGFA* vascular endothelial growth factor, *ESR1* estrogen receptor 1, *AdipoQ* adiponectin, *CD* cluster of differentiation, *PcF* pericellular fibrosis, *IL* interleukin, *TNF* tumor necrosis factor, *MCP* Monocyte Chemoattractant Protein. N = 26.

^a^Model corrected for total fat mass.

### Visceral adipose tissue phenotype and estimated insulin sensitivity

In VAT, increased HIF-1α and MCP-1 expression was associated with decreased estimated IS even after correcting for VAT mass (β = − 0.37, p = 0.01 and β = − 0.25, p = 0.03), whereas the associations between increased adipocyte size and PcF and estimated IS were only borderline significant after adjusting for VAT mass (β = − 0.14, p = 0.06 and β = − 0.36, p = 0.06) (Table 3). The regression analyses revealed that the association between VAT adipocyte size, PcF, and HIF-1α and estimated IS were stronger than the association between estimated IS and VAT mass itself (β_s adipocyte size_: − 0.415 vs. β_s VAT mass_: − 0.227; β_s PcF_: − 0.377 vs. β_s VAT mass_: − 0.349; β_s HIF-1α_: − 0.516 vs. β_s VAT mass_: − 0.504). Increased VAT IL-6 and TNF-α expression were also associated with decreased estimated IS, however, these associations were diminished after correcting for VAT mass (β = − 0.34, p = 0.16 and β = − 0.27, p = 0.08).

**Table 3:** Visceral adipose tissue inflammation and morphology associated with estimated insulin sensitivity.

	Estimated insulin sensitivity, AU^a^		Estimated insulin sensitivity, AU^a,b^	
	β (95% CI)	p-value	β (95% CI)	p-value
VAT adipocyte size	− 0.19 (− 0.31 to − 0.07)	0.004	− 0.14 (− 0.30 to 0.01)	0.06
VAT PPAR-γ	0.05 (− 0.57 to 0.67)	0.88	0.03 (− 0.71 to 0.78)	0.93
VAT FAS	0.49 (− 0.02 to 1.00)	0.06	0.10 (− 0.39 to 0.60)	0.67
VAT SCD1	0.16 (− 1.06 to 1.37)	0.79	0.23 (− 1.23 to 1.69)	0.75
VAT HIF-1α	− 0.32 (− 0.67 to 0.03)	0.07	− 0.37 (− 0.66 to − 0.09)	0.01
VAT VEGFA	0.03 (− 0.27 to 0.32)	0.86	0.03 (− 0.23 to 0.29)	0.82
VAT ESR1	0.05 (− 0.38 to 0.48)	0.80	− 0.09 (− 0.48 to 0.31)	0.65
VAT AdipoQ	0.06 (− 0.24 to 0.36)	0.69	0.06 (− 0.21 to 0.34)	0.63
VAT CD163^+^ cells	− 0.37 (− 1.01 to 0.28)	0.25	0.05 (− 0.67 to 0.77)	0.89
VAT CD68^+^ cells	0.25 (− 0.06 to 0.57)	0.11	0.24 (− 0.04 to 0.52)	0.09
VAT CD3^+^ cells	− 0.31 (− 0.84 to 0.22)	0.24	− 0.21 (− 0.71 to 0.28)	0.38
VAT CD20^+^ cells	− 0.33 (− 0.69 to 0.04)	0.08	− 0.17 (− 0.56 to 0.23)	0.39
VAT PcF	− 0.46 (− 0.82 to − 0.1)	0.02	− 0.36 (− 0.72 to 0.01)	0.06
VAT IL-6 mRNA	− 0.52 (− 0.96 to − 0.09)	0.02	− 0.34 (− 0.81 to 0.14)	0.16
VAT IL-18 mRNA	− 0.17 (− 0.35 to 0.01)	0.07	− 0.13 (− 0.29 to 0.04)	0.13
VAT TNF-α mRNA	− 0.35 (− 0.67 to − 0.03)	0.03	− 0.27 (− 0.57 to 0.04)	0.08
VAT MCP-1 mRNA	− 0.22 (− 0.59 to 0.15)	0.20	− 0.25 (− 0.47 to − 0.03)	0.03

*VAT* visceral adipose tissue, *AU* arbitrary units, *PPAR* Peroxisome proliferator activated receptor, *FAS* fatty-acid synthase, *SCD* Stearoyl-CoA desaturase, *HIF* Hypoxia-inducible factor, *VEGFA* vascular endothelial growth factor, *ESR1* estrogen receptor 1, *AdipoQ* adiponectin, *CD* cluster of differentiation, *PcF* pericellular fibrosis, *IL* interleukin, *TNF* tumor necrosis factor, *MCP* Monocyte Chemoattractant Protein. N = 25.

^a^Estimated insulin sensitivity calculated through Composite Matsuda Index.

^b^Model corrected for VAT mass.

Apart from a negative association between SAT CD3^+^ cell infiltration and estimated IS (β = − 0.08, p = 0.01), SAT phenotype was not associated with estimated IS (Supplementary Information 1).

### Aging

SAT inflammation and -morphology was not correlated to age. Increasing age was significantly associated with increased VAT fibrosis (R = 0.429, p = 0.02), CD163^+^ cell infiltration (R = 0.504, p = 0.005), IL-18 (R = 0.755, p = 0.005) and TNF-α expression (R = 0.463, p = 0.02) (Supplementary Information 2).

## Discussion

The key findings of this study were that postmenopause is associated with changes in AT phenotype related to metabolic dysfunction in both SAT and VAT. Whereas increased SAT inflammation in the context of menopause is associated with VAT accumulation, changes in VAT morphology is associated with decreased estimated IS.

Adipocyte hypertrophy was evident in both SAT and VAT of postmenopausal women and the number of adipocytes per kg fat was reduced with menopause. Even the calculated total number of adipocytes seemed to be 5–15% lower in the postmenopausal women, although this difference did not reach statistical significance. The fact that loss of endogenous sex hormone production is associated with adipocyte hypertrophy is in accordance with studies in rodents, where oophorectomy leads to an increase in adipocyte size in both inguinal and gonadal adipose depots^18–20^. The lower number of adipocytes in postmenopausal women indicate that a number of adipocytes might perish in relation to menopause. Estrogen has been shown to regulate cell death in several cell types^22,23^. However, whether the lower adipocyte number after menopause in this study reflects adipocyte apoptosis induced by estrogen withdrawal remains speculative. What drives the adipocyte hypertrophy with menopause is yet to be understood. In this study, we investigated the mRNA expression of a subset of adipogenic and lipogenic markers. We found the key rate-limiting enzyme in de novo lipogenesis, FAS, to be decreased in SAT of postmenopausal women. FAS expression has previously been shown to be lower with obesity^24^, and we speculate that the downregulation of FAS in this study could reflect a compensatory attempt to avoid further lipid deposition in the adipocyte. Inability to store excess lipids in SAT is known to cause lipid overflow and ectopic lipid deposition. In accordance with this, we found the low FAS expression in SAT to be strongly associated with increased VAT deposition. VAT PPAR-γ expression was increased in postmenopausal women which could reflect an attempt to overcome the increased need for lipid storage in this depot. However, this alone is unlikely to explain the substantial differences in VAT mass across menopause. Studies in rodents generally show increased mRNA expression of lipogenic and adipogenic markers in both inguinal and gonadal depots following oophorectomy^17,25,26^ but functional and mechanistic studies are highly warranted to understand how adipogenesis and lipogenesis are regulated with loss of ovarian function.

Adipocyte hypertrophy is believed to cause hypoxia contributing to the induction of AT inflammation^27,28^. In relation to this, we found that increased adipocyte size was associated with increased HIF-1α expression (the major mediator of the hypoxic response^28^) in both SAT and VAT. Furthermore, the HIF-1α promotor region is known to contain estrogen response elements and estrogen has been shown to ameliorate HIF-1α signaling to prevent AT hypoxia and fibrosis^29^. Consequently, both the increased adipocyte size and estrogen withdrawal could contribute to the vastly increased expression of HIF-1α in SAT after menopause. Postmenopausal women displayed increased PcF in both SAT and VAT. A previous study showed that increasing AT PcF was associated with a decreased ability to loose fat^15^. Thus, we speculate, that the pathologic AT remodeling could compromise the ability to lose weight, e.g. through exercise, following menopause. Estrogen has been suggested to prevent the production of reactive oxygen species (inducers of HIF-1α expression), deteriorate mitochondrial function, and counteract healthy adipose tissue expansion^30–33^. Taking this into consideration, future studies should investigate the possible role of reactive oxygen species in the relationship between loss of ovarian function and changes in adipose tissue phenotype.

Estrogen stimulates the expression of VEGFA (inducing neovascularization in the tissue to avoid hypoxia)^34^. Paradoxically, we showed that VEGFA expression in VAT was increased with menopause and propose that this could be a compensatory reaction to a hypoxic environment in the AT^35^. Adiponectin improves oxidative conditions and enhance insulin sensitivity^36,37^. Thus, we speculated that an increased expression of adiponectin in premenopausal adipose tissue could contribute to the beneficial metabolic phenotype in this group of women. However, we saw no differential expression of adiponectin in either SAT or VAT across menopause. This is in accordance with a previous study investigating circulating adiponectin levels in both humans and mice^38^.

The mechanisms underlying the redistribution of fat towards increased VAT deposition with menopause are unknown. In this study, increased expression of inflammatory markers, immune cell infiltration, hypoxia, and decreased expression of FAS in SAT were all associated with increased VAT deposition, even after correcting for total fat mass. This finding could suggest that subcutaneous AT dysfunction might contribute to VAT deposition following menopause. It is widely debated whether subcutaneous AT serves a protective role or contributes to metabolic deterioration and this is likely to depend on the anatomic location—as well as the morphologic phenotype of the subcutaneous adipose depot^8,39^. In relation to this, immune cell infiltration in the subcutaneous fat compartment have previously been associated with increased VAT mass in both humans and rodents and genetically modified rodent models where immune cell infiltration in AT are depleted are protected against IR^39–42^.

Low ESR1 expression in adipose tissue has been related to adiposity in humans^43^ and studies in rodents show that AT depletion of ESR1 leads to mitochondrial dysfunction, adipocyte hypertrophy and IR^29,43,44^. In this study, we found no differences in expression of ESR1 across menopausal status in either SAT or VAT indicating that it is not a differential expression of ESR1 per se that drives the menopausal differences in AT phenotype. As expected, serum follicle stimulating hormone (FSH) increased across menopause. A recent study in rodents suggest an independent effect of FSH on adiposity as blocking FSH reduced body fat^45^. This mechanism has not been demonstrated in human but could indicate that the metabolic deterioration with menopause might be driven through both low estrogen and high FSH.

We did not find any differences in circulating lipids across menopause, despite considerable differences in both SAT and VAT adipose morphology. However, in this study, we only assessed fasting plasma lipids. Thus, it is possible that a metabolic challenge, such as energy intake or insulin clamping, is necessary to detect menopausal differences in lipid flow in this, metabolically, relatively healthy group of middle-aged women.

Apart from increased VAT MCP-1 expression, VAT inflammation was not related to estimated IS after correcting for VAT mass, suggesting that it is unlikely to be VAT inflammation per se that drives the IR following menopause. Opposite to this, markers of VAT morphology (increased adipocyte size, -PcF, and -HIF-1α) tended to be associated with decreased estimated IS and the association between all three and estimated IS were stronger than the association between estimated IS and VAT mass itself. Neither SAT inflammation nor morphology was related to estimated IS. These findings are in accordance with a previous study in males, that found increased VAT adipocyte size, but not inflammation, to predict IR and further showed that SAT phenotype was unrelated to IR^46^. The fact that VAT, but not SAT, phenotype was associated with estimated IS might be explained by the anatomic differences in location of adipose depots, with the close proximity between the VAT depot and the liver, where lipid overflow is believed to cause hepatic lipotoxicity and thereby IR^47^. IR itself has also been shown to cause adipose tissue inflammation^48^. Thus, we cannot exclude that the IR could precede and promote the observed adipose tissue inflammation in postmenopausal women and the causal line of these actions should be investigated in future longitudinal studies.

Menopause was associated with increased CD163^+^ cell infiltration in VAT. CD163^+^ cells, originally referred to as M2 macrophages, are believed to play a role in tissue repair and matrix remodeling^49^. Total number of CD163^+^ cells in AT is known to increase with obesity and has been associated with IR^50^. Whether the increased CD163^+^ cell number is compensatory or contribute to AT inflammation remains debated^51,52^. In this study, we found no association between number of CD163^+^ cells, in either SAT or VAT, and estimated IS suggesting that the link between the two might be subsidiary. CD20^+^ cell number was also increased in VAT of postmenopausal women, reflecting an increased B-cell infiltration in the adipose depot. Studies in rodents suggest an important role for AT B-cells in the early stages of high-fat diet induced IR^42^.

Our study has some limitations. The study was based on cross-sectional data and consequently, causal conclusions could not be drawn. Furthermore, we cannot exclude, that differential indications for the gynecological surgeries could be related to metabolic phenotype and thereby confound the results as both uterine fibroids and heavy menstrual bleedings are more frequent with obesity^53,54^. However, types of surgeries were evenly distributed between pre- and postmenopausal women suggesting that the confounding effect was limited.

Five women were defined as perimenopausal and due to the limited size of this study population, results from the perimenopausal group should be interpreted with precaution. The inclusion of the small but complex group of perimenopausal women in the study, could impact the statistical comparisons between pre- and postmenopausal women. Thus, statistical analyses were explored without the perimenopausal group, with no major impact on the overall conclusions.

With the cross-sectional nature of the study, we cannot rule out, that especially women in the perimenopausal group had had their last menstrual period and would therefore be allocated to the postmenopausal group if sampled shortly after.

Postmenopausal women were approximately 5 years older than premenopausal women. Studies in rodents show, that aging is associated with increased inflammation in gonadal AT^55–57^. In accordance with this, we showed that high age to some extent was associated with increased VAT inflammation. Due to the multicollinear nature of menopause and age, analyses were not adjusted for age. However, the age span in this study was relatively narrow and the effects of age on adipose phenotype, especially in SAT, were sparse. Thus, the contribution of age to the overall findings are believed to be modest.

We conclude that menopause is associated with changes in AT phenotype related to metabolic dysfunction in both SAT and VAT. Whereas increased SAT inflammation in the context of menopause was associated with VAT accumulation, changes in VAT morphology was associated with decreased estimated IS. Based on these findings, we speculate that changes in SAT and VAT phenotype in relation to menopause could represent important mechanistic links between menopause, adipose redistribution and IR. Furthermore, the findings suggest a metabolically more detrimental effect of expanding the SAT and VAT depots after menopause. Thus, future studies should focus on efficient strategies such as diet or exercise to prevent postmenopausal metabolic deterioration and adipose tissue dysfunction.

## Methods

### Participants

Women between 45 and 60 years were recruited through the Department of Gynecology, Rigshospitalet, Denmark, and were all undergoing benign gynecological surgeries. Types of surgeries included hysterectomy, and uni- and bilateral oophorectomies. Included indications for hysterectomy were uterine fibroids and menstrual bleeding disorders. Indications for unilateral oophorectomies were ovarian cysts and bilateral oophorectomies were performed due to genetic predisposition to cancer.

The women were divided into premenopausal, perimenopausal and postmenopausal based on menstrual status and FSH. In accordance with international guidelines, a period of 12 months of amenorrhea is a prerequisite for the label of postmenopause^58^. A cut-off of FSH = 20 IU/l were chosen to distinguish a regularly ovulating ovary from an ovary with a gradually declining function, in accordance with larger population studies^59^.

Premenopausal women were defined as having regular menstrual bleedings (10 menstrual bleeding within the last 12 months) and serum FSH < 20 IU/l. Postmenopausal as having amenorrhea for > 12 months and FSH > 20 IU/l. Women who did not meet the subdivision criteria because of menstrual bleedings within the last 12 months despite serum FSH > 20 IU/l were defined as perimenopausal.

Exclusion criteria were: (1) chronic diseases (including endometriosis), (2) infections during the last 4 weeks, (3) more than 7 alcohol units/week, (4) premature menopause (before age 40 years), (5) hysterectomy or oophorectomy prior to study inclusion, (6) body mass index (BMI) > 35 kg/m^2^ (as MRIs could not be performed with higher BMI).

Premenopausal women with regular menstrual bleedings went through all tests in the follicular phase of their menstrual cycle (day 1–8). Women with irregular bleedings were tested on a random cycle day, where serum FSH were obtained simultaneously. Postmenopausal women were also tested on a random day.

A subset of the women (n = 18) also participated in a sub-study investigating ectopic lipid deposition with menopause and systemic inflammation, published previously^4,21^.

Informed consent was obtained in writing and verbally from all participants. The study was approved by the ethical committee of the Capital Region, Denmark (H-3-2014-096) and performed according to the declaration of Helsinki.

### Study design

A maximum of 1 month prior to surgery, women reported to the laboratory in the morning, on two separate days, following an overnight fast. All women were instructed not to perform any vigorous exercise 48 h prior to the experiment and abstain from coffee, tea, or alcohol 24 h before. The first experimental day included (1) health exam, and (2) oral glucose tolerance test (OGTT). The second day included (1) whole-body dual-energy X-ray absorptiometry (DXA) scan, (2) biopsies from the SAT and skeletal muscle, and (3) fitness test (VO_2_ max test).

On the day of surgery, a biopsy was collected from the omentum majus when surgery was initiated. All biopsies (including omental) were taken before noon, following an overnight fast.

### Body composition

Fat- and lean body masses were assessed through a DXA scan (Lunar Prodigy Advance; GE Medical Systems Lunar, Milwaukee, WI). Software (Prodigy, enCORE 2004, version 8.8; GE Lunar Corp, Madison, WI) was used to estimate regional- and total fat, and fat-free tissue masses.

Magnetic resonance imaging (MRI) of the abdomen was performed using a Siemens Magnetom Prisma 3 Tesla matrix magnetic resonance scanner (Erlangen, Germany) at 3-mm intervals as described previously^4^. All adipose tissue located from the diaphragm to pelvic floor inside the peritoneum was traced manually as the visceral fat region of interest. Multi-image analysis software^60^ (Mango; Research Imaging Institute, Houston, TX) was used to calculate the total volume of visceral fat from the T1-weighted MRI sequence. A single reader, who was blinded to the menopausal status of the subjects, performed all image analyses.

### Estimated insulin sensitivity

Whole-body estimated IS was calculated from an OGTT using the Composite Matsuda IS Index^61^. Blood samples were drawn at time points − 10, 0, 15, 30, 60, 90, and 120 min after drinking 83 g of glucose monohydrate (corresponding to 75 g anhydrous glucose) dissolved in 300 mL of water^62^. Baseline values were calculated as a mean of the − 10- and 0-min samples.

### Adipose tissue biopsies

Biopsies from abdominal SAT were obtained with a modified Bergström needle. VAT biopsies were collected as soon as surgery was initiated. All AT biopsies were divided in three; (1) frozen in liquid nitrogen and stored at − 80 °C, (2) fixed in 2% depolymerized paraformaldehyde plus 0.15% picric acid and embedded in paraffin, (3) isolation of adipocyte precursors.

### Immunohistochemistry

Paraffin-embedded biopsies were sectioned (5 μm thick) in two depths at least 200 µm apart.

Details on immunohistochemistry staining and antibodies are shown in Supplementary Information 3.

Stained cells were counted manually by a single person blinded to the menopausal status of the subjects. In order to get a representative sampling at least 100 adipocytes were counted for each ID^63^. Cells of interest were counted in at least two different regions of the embedded tissue. Quantification of cells was performed using ImageJ software (NIH, Bethesda, MD, USA).

Median volume of adipocytes was estimated using the formula V = 4/3·π·r^2^, and total number was calculated dividing the volume of the fat depot with median volume of adipocytes.

### RNA isolation and quantitative real-time PCR

Total RNA was extracted from AT using RNeasy lipid tissue mini kit (Qiagen, Hilden, Germany). RNA was reverse transcribed according to the manufacturer’s protocol (Applied Biosystems, Foster City, CA, USA). The RNA levels were determined by quantitative real time PCR using a ViiA 7 sequence detector (Applied Biosystems, Foster City, CA, USA). A mean value of 18S rRNA and GAPDH mRNA levels were used as endogenous control. Primer sequences are shown in Supplementary Information 4.

Due to limited tissue, five pre- and five postmenopausal women were not included in SAT PCR analyses. This was the case for two pre- and two postmenopausal women in VAT PCR analyses.

### ***VO***_***2***_*** max test***

VO_2_ max was determined using a progressive exercise test on a bicycle ergometer (Monark 839E, Monark, Varberg, Sweden) with a 5-min warm up at 70 watts followed by a 20-W increase in workload each minute until exhaustion. VO_2_ was continuously measured by indirect calorimetry (Quark b2, Cosmed, Rome, Italy).

### Questionnaires

Participants completed a Minnesota Leisure Time Physical Activity Questionnaire (MLTPAQ) to determine their physical activity levels over the last 4 months, estimated as an activity metabolic index^64^. The daily energy intake was estimated from a comprehensive 3-day food diary before the first visit. Based on this, mean caloric intake was calculated.

### Statistics

All variables were log-transformed if not normally distributed. Data are presented as median (IQR) unless otherwise stated. Variables of interest were compared using a one-way ANOVA with a Fisher’s Least Significance Difference post-hoc test. Regression models were checked for assumptions of the linear model, including normal distribution of the residuals, homogeneity of variance, linearity, and independent observations. Standardized β (β_S_) was used to estimate the contribution of each predictor to the dependent variable, within a multiple regression analysis. Distribution of adipocyte size was assessed using a mixed model. A random effect accounting for an individual specific level was included.

Simple correlations between variables were investigated using Pearson correlation analyses.

Mixed model analyses were performed in SAS Enterprise Guide version 7.1. Other statistical analyses were performed using IBM SPSS statistics version 25. P < 0.05 was considered statistically significant.

## Supplementary Information

Supplementary Information.

## Data Availability

The datasets generated during and/or analyzed during the current study are not publicly available due to Danish legislation on data protection but are available from the corresponding author on reasonable request and with permission from the Ethical Committee of the Capital Region, Denmark.
