# Supplementary material for: Changes in abdominal subcutaneous adipose tissue phenotype following menopause is associated with increased visceral fat mass
Source: Sci Rep. 2021 Jul 20;11:14750. doi: 10.1038/s41598-021-94189-2 (PMC8292317; doi:10.1038/s41598-021-94189-2)
Supplement: Supplementary file 1 — Supplementary Information. [file 41598_2021_94189_MOESM1_ESM.pdf]

Supplementary Information

Changes in abdominal subcutaneous adipose tissue phenotype following menopause is associated with increased visceral fat mass

Julie Abildgaard[1,2*], Thorkil Ploug[3], Elaf Al-Saoudi[1], Thomas Wagner[4], Carsten Thomsen[5], Caroline Ewertsen[6], Michael Bzorek[7], Bente Klarlund Pedersen[1], Anette Tønnes Pedersen[8], Birgitte Lindegaard[1,9]

[1] The Centre of Inflammation and Metabolism and the Centre for Physical Activity Research, Rigshospitalet, University of Copenhagen, Copenhagen, Denmark
[2] Department of Growth and Reproduction, Copenhagen University Hospital, Rigshospitalet, Copenhagen, Denmark
[3] Department of Biomedical Sciences, Faculty of Health and Medical Sciences, University of Copenhagen, Copenhagen, Denmark
[4] Department of Oncology, Copenhagen University Hospital, Rigshospitalet, Copenhagen, Denmark
[5] Department of Radiology, Zealand University Hospital, Roskilde, Denmark
[6] Department of Radiology, Copenhagen University Hospital, Rigshospitalet, Copenhagen, Denmark
[7] Department of Pathology, Zealand University Hospital, Næstved, Denmark
[8] Department of Gynecology, Copenhagen University Hospital, Rigshospitalet, Copenhagen, Denmark
[9] Department of Pulmonary and Infectious Diseases, Nordsjællands University Hospital, Hillerød, Denmark

Supplementary 1. Subcutaneous adipose tissue inflammation and morphology associated with estimated insulin sensitivity.

	**Estimated insulin sensitivity, AU[a]**		**Estimated insulin sensitivity, AU[a,b]**	
	β (95 % CI)	p-value	β (95 % CI)	p-value
SAT adipocyte size	-0.07 (-0.79 – 0.64)	0.83	1.66 (-0.48 – 0.82)	0.60
SAT PPAR-γ	0.46 (-0.41 – 1.33)	0.28	0.16 (-0.76 – 1.08)	0.72
SAT FAS	0.26 (-0.06 – 0.58)	0.09	0.15 (-0.21 – 0.52)	0.39
SAT SCD1	0.26 (-0.31 – 0.82)	0.35	0.13 (-0.43 – 0.68)	0.64
SAT HIF-1α	0.06 (-0.45 – 0.57)	0.81	0.25 (-0.24 – 0.74)	0.30
SAT VEGFA	0.29 (-0.04 – 0.62)	0.08	0.19 (-0.18 – 0.56)	0.29
SAT ESR1	0.22 (-0.10 – 0.53)	0.16	0.10 (-0.26 – 0.46)	0.56
SAT AdipoQ	0.26 (-0.04 – 0.55)	0.08	0.14 (-0.24 – 0.53)	0.44
SAT CD163$^+$ cells	-0.32 (-0.67 – 0.02)	0.06	-0.05 (-0.53 – 0.43)	0.83
SAT CD68$^+$ cells	-0.31 (-0.71 – 0.10)	0.13	-0.27 (-0.62 – 0.09)	0.13
SAT CD3$^+$ cells	-0.62 (-1.00 – -0.25)	0.003	-0.51 (-0.86 – -0.16)	0.007
SAT CD20$^+$ cells	-0.27 (-0.65 – 0.10)	0.14	-0.12 (-0.49 – 0.26)	0.52
SAT PcF	-0.55 (-0.96 – -0.13)	0.01	-0.37 (-0.82 – 0.08)	0.10
SAT IL-6 mRNA	-0.20 (-0.71 – 0.31)	0.42	0.01 (-0.54 – 0.56)	0.98
SAT IL-18 mRNA	-0.31 (-0.79 – 0.18)	0.20	-0.08 (-0.67 – 0.52)	0.79
SAT TNF-α mRNA	-0.05 (-0.50 – 0.41)	0.83	0.05 (-0.38 – 0.48)	0.81
SAT MCP-1 mRNA	-0.04 (-1.02 – 0.94)	0.93	0.12 (-0.80 – 1.04)	0.79

[a] Estimated insulin sensitivity calculated through Composite Matsuda Index.
[b] Model corrected for total mass
SAT, subcutaneous adipose tissue. PPAR, Peroxisome proliferator activated receptor. FAS, fatty-acid synthase. SCD, Stearoyl-CoA desaturase. HIF, Hypoxia-inducible factor. VEGFA, Vascular endothelial growth factor A. ESR1, Estrogen receptor 1. AdipoQ, adiponectin. CD, cluster of differentiation. PcF, pericellular fibrosis. IL, interleukin. TNF, tumor necrosis factor. MCP, Monocyte Chemoattractant Protein.

Supplementary 2. Adipose tissue inflammation and morphology correlated to age

Age		
	R	p-value
SAT adipocyte size	-0.026	0.90
VAT adipocyte size	0.306	0.10
SAT PPAR-γ	-0.038	0.87
VAT PPAR-γ	0.048	0.81
SAT FAS	-0.283	0.21
VAT FAS	-0.288	0.15
SAT FAS	-0.276	0.23
VAT FAS	0.056	0.78
SAT fibrosis	0.330	0.10
VAT fibrosis	0.429	0.02
SAT CD163$^+$ cell infiltration	0.396	0.06
VAT CD163$^+$ cell infiltration	0.504	0.005
SAT CD68$^+$ cell infiltration	0.032	0.88
VAT CD68$^+$ cell infiltration	-0.006	0.98
SAT CD3$^+$ cell infiltration	0.172	0.43
VAT CD3$^+$ cell infiltration	0.243	0.21
SAT CD20$^+$ cell infiltration	0.126	0.55
VAT CD20$^+$ cell infiltration	0.290	0.13
SAT IL-6	0.348	0.12
VAT IL-6	0.220	0.27
SAT IL-18	0.533	0.17
VAT IL-18	0.755	0.005
SAT TNF-α	0.012	0.96
VAT TNF- α	0.463	0.02
SAT MCP-1	0.236	0.30
VAT MCP-1	0.169	0.40
SAT HIF-1α	0.209	0.36
VAT HIF-1α	0.278	0.16

SAT, subcutaneous adipose tissue. VAT, visceral adipose tissue. PPAR, Peroxisome proliferator activated receptor. FAS, fatty-acid synthase. SCD, Stearoyl-CoA desaturase. HIF, Hypoxia-inducible factor. VEGFA, Vascular endothelial growth factor A. ESR1, Estrogen receptor 1. CD, cluster of differentiation. PcF, pericellular fibrosis. IL, interleukin. TNF, tumor necrosis factor. MCP, Monocyte Chemoattractant Protein.

Supplementary 3. Immunohistochemistry methods and antibody details

Methods

Dewaxing and antigen retrieval was performed by immersing slides in EnVision™ FLEX Target Retrieval Solution, High pH (Dako, Glostrup, cat.no K8004) and heated at 65ºC for 16 hours in the PT-module according to the manufacturer's instructions. After pre-treatment, slides were incubated with the primary antibodies for 30 min. The reactions were detected/visualized using the standard polymer technique EnVision™ FLEX /HRP Detection Reagent (Dako, cat. no K8000) and signal intensity was enhanced using EnVision™ FLEX+ Mouse (LINKER) (Dako, cat. no K8021). Finally, sections were counterstained with hematoxylin and mounted with pertex.

Hematoxylin-eosin staining was used as counter-staining and furthermore used to quantify adipocyte size.

Antibody	Clone	Manufacturer	Catalogue no.	Dilution
CD163	MRQ-26	Cell Marque, Rocklin, CA, USA	163M-16	1:25
CD68	PGM1	DAKO, Glostrup, Denmark	GA613	Ready-to-use
CD3	LN10	Leica Biosystem, Wetzlar, Germany	NCL-L-CD3-565	1:25
CD20	L26	DAKO, Glostrup, Denmark	GA604	Ready-to-use

CD, cluster of differentiation.

Supplementary 4. Primer sequences

	Primer sequence
**IL-6**	
Forward	5’ -TTTTGTACTCATCTGCACAGC-3’
Reverse	5’ -GGATTCAATGAGGAGACTTGC-3’
**IL-18**	
Forward	5’ -CAACAAACTATTTGTCGCAGCA -3’
Reverse	5’ -TGCCACAAAGTTGATGCAAT -3’
**TNF-α**	
Forward	5’ -TTGAGGGTTTGCTACAACATGGG-3’
Reverse	5’ -GCTGCACTTTGGAGTGATCG-3’
**MCP-1**	
Forward	5’-GTCTTGAAGATCACAGCTTCTTTGG-3’
Reverse	5’-AGCCAGATGCAATCAATGCC-3’
**HIF-1α**	
Forward	5’-CCCCAGTCACCTGCTGTTAT-3’
Reverse	5’-AGATCTCCTTGGCCACAATG-3’
**PPAR-γ**	
Forward	GAAACTTCAAGAGTACCAAAGTGCAA
Reverse	AGGCTTATTGTAGAGCTGAGTCTTCTC
Probe	FAM 5'-CAAAGTGGAGCCTGCATCTCCACCTTATT-3' Tamra
**FAS**	
Forward	5’-TGGTCACGGACGATGACCGTC-3’
Reverse	5’-GGTTGATGCCTCCGTCCACGAT-3’
**SCD1**	
Forward	5’-CACCCAGCTGTCAAAGAGAAGG-3’
Reverse	5’-AGGACGATATCCGAAGAGGTGG-3’
**VEGFA**	
Forward	5’- ATCTGCATGGTGATGTTGGA -3’
Reverse	5’- GGGCAGAATCATCACGAAG -3’
**ESR1**	
Forward	5’- AGGTGGACCTGATCATGGAG -3’
Reverse	5’- AAGCTTCGATGATGGGCTTA -3’
**AdipoQ**	
Forward	5’- AAAACCTCCCCCAAGCAGAGCTTC -3’
Reverse	5’- TGAGGAACAGGGATGAGTTCAGCA -3’

PPAR, Peroxisome proliferator activated receptor. FAS, fatty-acid synthase. SCD, Stearoyl-CoA desaturase. HIF, Hypoxia-inducible factor. VEGFA, Vascular endothelial growth factor A. ESR1, Estrogen receptor 1. AdipoQ, Adiponectin. IL, interleukin. TNF, tumor necrosis factor. MCP, Monocyte Chemoattractant Protein.
